# Turning the hands of time again: a purely confirmatory replication study and a Bayesian analysis

**DOI:** 10.3389/fpsyg.2015.00494

**Published:** 2015-04-24

**Authors:** Eric-Jan Wagenmakers, Titia F. Beek, Mark Rotteveel, Alex Gierholz, Dora Matzke, Helen Steingroever, Alexander Ly, Josine Verhagen, Ravi Selker, Adam Sasiadek, Quentin F. Gronau, Jonathon Love, Yair Pinto

**Affiliations:** ^1^Psychological Methods, University of Amsterdam, AmsterdamNetherlands; ^2^Social Psychology Program, University of Amsterdam, AmsterdamNetherlands; ^3^Brain and Cognition Center, University of Amsterdam, AmsterdamNetherlands; ^4^Cognitive Neuroscience Group, University of Amsterdam, AmsterdamNetherlands

**Keywords:** preregistration, replication, Bayes factor, statistical evidence, null hypothesis testing

## Abstract

In a series of four experiments, Topolinski and Sparenberg (2012) found support for the conjecture that clockwise movements induce psychological states of temporal progression and an orientation toward the future and novelty. Here we report the results of a preregistered replication attempt of Experiment 2 from Topolinski and Sparenberg (2012). Participants turned kitchen rolls either clockwise or counterclockwise while answering items from a questionnaire assessing openness to experience. Data from 102 participants showed that the effect went slightly in the direction opposite to that predicted by Topolinski and Sparenberg (2012), and a preregistered Bayes factor hypothesis test revealed that the data were 10.76 times more likely under the null hypothesis than under the alternative hypothesis. Our findings illustrate the theoretical importance and practical advantages of preregistered Bayes factor replication studies, both for psychological science and for empirical work in general.

## Introduction

**FIGURE 1:**
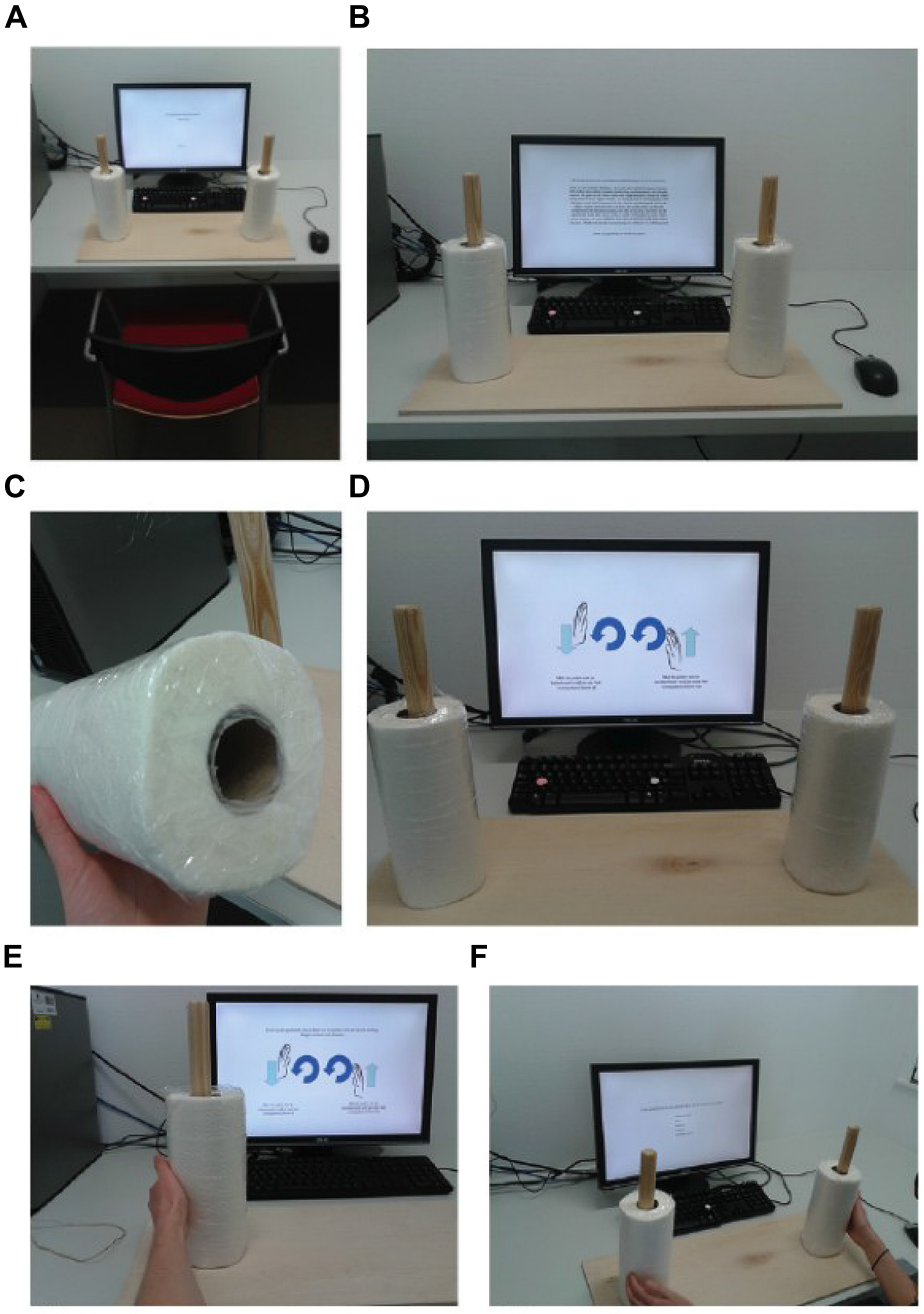
**The experimental setting: (A) the set-up; (B) the instructions; (C) a close-up of one of the sealed paper towels; (D) the schematic instructions; photos (E), and (F) give an idea of how a participant performs the experiment**. Figure available at: https://www.flickr.com/photos/130759277@N05/, under CC license https://creativecommons.org/licenses/by/2.0/.

In a series of four experiments, Topolinski and Sparenberg (2012) sought to demonstrate that clockwise movements induce psychological states of temporal progression and an orientation toward the future and novelty. For instance, participants in their Experiment 2 had to turn kitchen rolls either clockwise or counterclockwise (see **Figure 1**); as the authors predicted, the results showed that participants who turned the rolls clockwise reported more “openness to experience” than those that turned the rolls counterclockwise.

Recently, however, Francis (2013, p. 162) subjected the Topolinski and Sparenberg (2012) experiments to a test for publication bias and concluded that “The probability of the experiment set is low enough to conclude that the reported results appear inconsistent, which suggests that either the experiments were not fully reported or were not run properly.” We believe it is important to attempt and replicate the Topolinski and Sparenberg (2012) results, for several reasons. First, the effect may strike many researchers as implausible – hence, additional independent strictly confirmatory replication is needed to establish the effect more firmly (in case it exists). Second, the publication test by Francis (2013) casts doubt on the veracity of the set of experiments – it is therefore important to address this emerging debate by empirical means. Third, the theory, experimental design, and apparatus are simple and elegant; this is esthetically appealing and, in addition, the straightforward nature of the hypothesis and the experiment facilitates the design and analysis of a rigorous replication attempt. Fourth, the Topolinski and Sparenberg (2012) replication attempt allows us to highlight the advantages of preregistration in combination with Bayesian hypothesis tests.

In replication studies is it essential to be able to quantify evidence in favor of the null hypothesis. In addition, it is desirable to collect data until a point has been proven or disproven. Neither desideratum can be accomplished within the framework of frequentist statistics, and this is why our analysis of both experiments will focus on hypothesis testing using the Bayes factor (e.g., Edwards et al., 1963; Berger and Mortera, 1999; Wagenmakers, 2007; Wagenmakers et al., 2012; Rouder et al., 2012). The method section below provides the details of the proposed design and analysis methodology.

## Experiment

We sought to replicate Topolinski and Sparenberg (2012) Experiment 2: the kitchen roll experiment. This research followed a strictly confirmatory protocol as described in Wagenmakers et al. (2012) and advocated by De Groot et al. (1956/2014) and Chambers (2013) – the preregistration form is available on the Open Science Framework at https://osf.io/p3isc/. In line with the stipulations of this *Frontiers* special issue, the preregistration form was peer-reviewed and accepted before any data had been collected^1^ (cf. Chambers, 2013). The data are freely available at https://osf.io/uszvx/.

### Methods

#### Intended Sampling Plan

A traditional frequentist analysis would start with an assessment of effect size followed by a power calculation that seeks to determine the number of participants that yields a specific probability for rejecting the null hypothesis when it is false. This frequentist analysis plan is needlessly constraining and potentially wasteful: the experiment cannot continue after the planned number of participants has been tested, and it cannot stop even when the data yield a compelling result earlier than expected (e.g., Wagenmakers, 2007). We circumvented these limitations by calculating and monitoring the Bayes factor (e.g., Edwards et al., 1963; Berger and Mortera, 1999; Rouder et al., 2012; Wagenmakers et al., 2012). For the interpretation of evidence in the Bayesian paradigm, the intention with which the data are collected is irrelevant; hence, the Bayes factor can be monitored as the data come in, and data collection can be terminated at any point (Berger and Wolpert, 1988; Rouder, 2014).

Based on the above considerations, our sampling plan was as follows: we planned to collect data from a minimum of 20 participants in each between-subject condition (i.e., the clockwise and counterclockwise condition, for a minimum of 40 participants in total). We were then planning to monitor the Bayes factor and stop the experiment whenever the critical hypothesis test (detailed below) reached a Bayes factor that can be considered “strong” evidence (Jeffreys, 1961); this means that the Bayes factor is either of 10 in favor of the null hypothesis, or 10 in favor of the alternative hypothesis. We also planned to stop the experiment whenever we reached the maximum number of participants, which we set to 50 participants per condition (i.e., a maximum of 100 participants in total). Finally, we planned to stop the experiment on October 1st, 2013. From a Bayesian perspective the specification of this sampling plan is needlessly precise; we nevertheless felt the urge to be as complete as possible.

#### Intended Analyses

We planned to exclude from analysis those participants who discerned the goal of the experiment (e.g., “the experiment is about how personality changes due to turning kitchen rolls clockwise or counterclockwise”). The intended analysis proceeds as in Topolinski and Sparenberg (2012): we planned to recode the reverse items and then average the scores on the 12 openness to experience items for each participant. Then we planned to use a Bayesian hypothesis test to quantify the evidence for the hypothesis that participants who turn the kitchen rolls clockwise report higher openness to experience than participants who turn the rolls counterclockwise.

Specifically, we planned to assess this hypothesis by means of a default Bayes factor for an unpaired, one-sided *t*-test as outlined in Rouder et al. (2009) and Wetzels et al. (2009). Bayes factors quantify the support that the data provide for the null hypothesis vis-a-vis the alternative hypothesis. Support in favor of the alternative hypothesis constitutes support in favor of the effect reported by Topolinski and Sparenberg (2012) in their Experiment 2.

#### Deviations from OSF Preregistration Document

We deviated from the preregistration document in three aspects. The first aspect is that the preregistration document specified that we would recruit “Psychology students from the University of Amsterdam” (Wagenmakers et al., 2013). In practice, we did not. People interested in participation could make an appointment via the UvA-participant website.^2^ It turned out that this website is open to all UvA students (and not only psychology students as we initially assumed). Therefore, students from other academic fields also participated in the study. Also, seven participants made an appointment on site and were not students. As a result, our sample is more diverse compared to our initial sampling plan that only included UvA psychology students. There does not appear to be a compelling explanation for why the slightly more heterogeneous sample should materially change the outcome of the experiment, and hence we analyzed the data of all participants.

**FIGURE 2:**
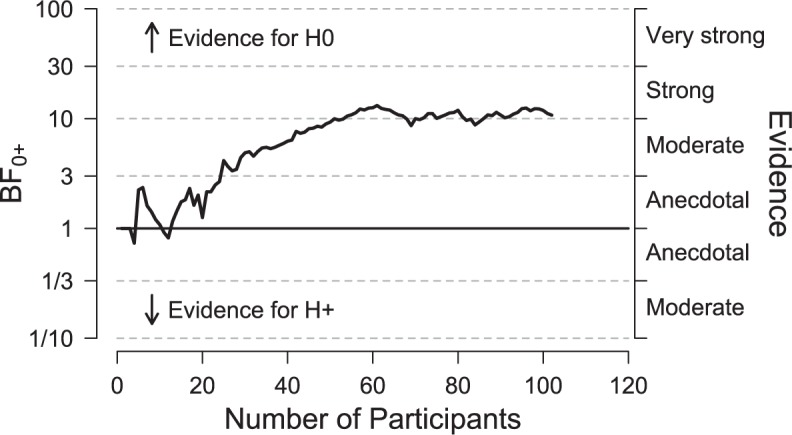
**The development of the Bayes factors for the comparison of openness to experience between clockwise and counter-clockwise rotation**. The end result is based on 48 participants in the clockwise condition and 54 participants in the counterclockwise condition. Figure adjusted from JASP, jasp-stats.org.

The second aspect in which we deviated from our protocol concerns the stopping rule; we planned to stop collecting data after obtaining a Bayes factor of 10 in favor of the null hypothesis, or 10 in favor of the alternative hypothesis. As can be seen in **Figure 2**, we reached this criterion several times before actually stopping. This occurred because data had to be entered into the analysis by hand and this made it more difficult to monitor the Bayes factor continually. In practice, the Bayes factor was checked every few days. Thus, we continued data collection until we reached our predetermined stopping criterion at the point of checking.

The final aspect in which we deviated from our protocol is that we tested 102 participants, which is more than the 100 that were planned initially. This deviation occurred because participants were randomly assigned to conditions (i.e., by picking an envelope that contained the number of their booth, see below). Hence, the main criterion of a maximum number of 50 participants per condition is not necessarily consistent with the secondary criterion of a maximum number of 100 participants total, as was assumed in the preregistration document. At the point of stopping, there were 48 participants in the clockwise condition and 54 in the counterclockwise condition.

#### Participants

As mentioned above, we recruited students from the University of Amsterdam as well as non-students (people who walked in). Participants were rewarded with course credits or a small monetary reward.

#### Materials

We closely followed the materials section in Topolinski and Sparenberg (2012; Experiment 2). Specifically, we used a shortened 12-item version of the openness to experience subscale of the Neuroticism–Extroversion–Openness Personality Inventory (NEO PI-R; Costa and McCrae, 1992), assessing a preference for novel experiences and actions. An example item includes “I often try new and foreign food.” As to-be-rotated objects, two ordinary kitchen paper towels were sealed with plastic and slipped loosely over upright wooden rods (see **Figures 1A,B**). Although this was not specified by Topolinski and Sparenberg (2012), we also sealed the bottom part of the paper towels, because this improved the ease of rotation (see **Figure 1C**).

Both rods were fixed on a wooden board, 50 cm apart, so that the two paper towels could easily be manipulated using both arms. The rotating direction was instructed non-verbally by a schematic description (**Figures 1D,E**). The above description copied Topolinski and Sparenberg (2012) almost to the letter, the only difference being that we used a translated, Dutch version of the 12-item NEO PI-R (Hoekstra et al., 1996). Other instructions with respect to the goal of the task and the turning of the kitchen rolls were directly translated from those employed by Topolinski and Sparenberg (2012) after Dr. Topolinski kindly provided us with the materials.

#### Procedure

After signing an informed consent form, participants first completed a set of unrelated tasks lasting approximately 30 min (e.g., completing an assessment form, doing a lexical decision task). This setup was used on purpose, as it mimicked more closely the design of Topolinski and Sparenberg (2012), where the kitchen roll task was also presented following a battery of other, unrelated tasks (this was pointed out to us by Dr. Topolinski).

Next, we closely followed the procedures outlined by Topolinski and Sparenberg (2012): participants were instructed to continually rotate the two paper towels using the palms of their hands (**Figures 1D,E**); this activity was practiced for 75 s, after which participants used the mouse to evaluate the pleasantness and effort of rotating the rolls. The experiment then started: participants rotated the rolls while reading items from the personality questionnaire, stopping to rotate only to use the computer in order to complete the items. To reduce across-participant variability in rotation times and consequently increase the probability of finding an effect, we implemented a 10-s rotation period preceding each question (this deviates from Topolinski and Sparenberg, 2012, who did not implement such a rotation period and responding was self-paced). Participants used the mouse to render their ratings on a 5-point scale from -2 (*strongly disagree*) to +2 (*strongly agree*). Then, participants were asked to report their current mood (0 = *very bad* and 10 = *very good*) and arousal (0 = *very relaxed* and 10 = *very aroused/excited*) while still rotating. Crucially, the turning direction of the paper towels was either clockwise or counterclockwise; participants were assigned to each of these conditions in random fashion.

As in Topolinski and Sparenberg (2012), all rating scales are presented vertically (see **Figure 1F**). A minor design change with respect to Topolinski and Sparenberg (2012) is that, within each condition of rotation, we counterbalanced the ordering of the options: half of the participants saw the lowest-rated options at the bottom of the screen, and half of the participants saw the lowest-rated options at the top of the screen.

As in Topolinski and Sparenberg (2012), we attempted to minimize the interaction between the participants and the experimenter. Hence, participants completed the task in individual subject booths; the doors to the booths remained open so that the experimenter could unobtrusively confirm that the participants were turning the rolls as instructed. Assignment of participants to conditions was determined by the random draw of an envelope that contained the number of the booth, and occurred immediately prior to the participant entering the booth. In case a participant was confused about the instructions the experimenter briefly provided clarification. After responding to the last item, a PC-directed funneled debriefing probed for participants’ suspicions concerning the purpose of the experiment.

## Results

### Exclusion of Participants

We excluded five participants who did not follow the experimental procedure as intended: two of these participants rotated two rolls in the opposite direction (e.g., with the left hand clockwise and with the right hand counter-clockwise), one participant stopped rotating after the first NEO-item, one participant misunderstood the instructions and tried to rotate the wooden sticks instead of the rolls, and one participant expressed strong dissatisfaction with the task (consequently, the experimenter decided to stop the task halfway).

We included a total of 102 participants (77 females) in the analysis, 48 in the clockwise condition and 54 in the counterclockwise condition. The mean age was 22.1 years (range 17–51) and 93% (*N* = 95) participants were students.

### Confirmatory Analysis

We recoded the reverse items (Cronbach’s α = 0.65, similar to the value of α = 0.58 reported in Topolinski and Sparenberg, 2012) and then averaged the scores on the 12 openness to experience items for each participant. We used a one-sided Bayesian hypothesis test (with a default Cauchy prior width of *r* = 1 for effect size on the alternative hypothesis, as specified by Rouder et al., 2009 and Wetzels et al., 2009) to quantify the evidence for the hypothesis that participants who turn the kitchen rolls clockwise report higher openness to experience than participants who turn them counterclockwise. The Bayes factor is BF_01_ = 10.76, indicating that the observed data are 10.76 times more likely under the null hypothesis that postulates the absence of the effect than under the alternative hypothesis that postulates the presence of the effect. According to the classification scheme proposed by Jeffreys (1961), this is strong evidence for the null hypothesis that there is no difference on the NEO between people rotating clockwise vs. counterclockwise. The mean score on the Openness to Experience items was smaller in the clockwise condition (*Mean* = 0.64, SD = 0.50, *N* = 48) than in the counterclockwise condition (*Mean*= 0.71, SD = 0.47, *N* = 54); hence, the observed effect goes slightly in the direction opposite to that reported by Topolinski and Sparenberg (2012).

### Exploratory Analysis 1: Development of the Bayes Factor

**Figure 2** shows the development of the Bayes factor as a function of the number of participants that were tested. Values of BF_0+_ greater than 1 indicate evidence in favor of the null hypothesis. As the number of participants grows, the Bayes factor increasingly supports the null hypothesis. It is of note that this Bayesian sequential analysis requires no corrections – the Bayes factor can simply be monitored as the data accumulate (e.g., Edwards et al., 1963; Berger and Mortera, 1999; Wagenmakers et al., 2012).

### Exploratory Analysis 2: Posterior Distribution on Effect Size

**FIGURE 3:**
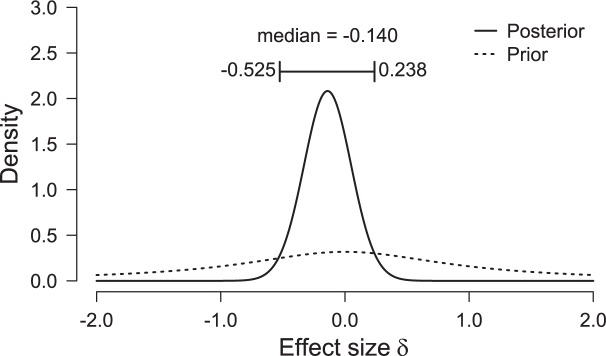
**Posterior distribution of effect size under a two-sided prior distribution (Rouder et al., 2009)**. Most posterior mass is to the left of zero, consistent with the fact that –in contrast to Topolinski and Sparenberg (2012) – openness to experience scores are lower in the clockwise condition than in the counterclockwise condition. Figure adjusted from JASP, jasp-stats.org.

**Figure 3** shows the posterior distribution for effect size (based on a two-sided prior distribution). Most posterior mass is to the left of zero, consistent with the fact that –in contrast to Topolinski and Sparenberg (2012)– openness to experience scores are lower in the clockwise condition than in the counterclockwise condition.

### Exploratory Analysis 3: Assessing Robustness

**FIGURE 4:**
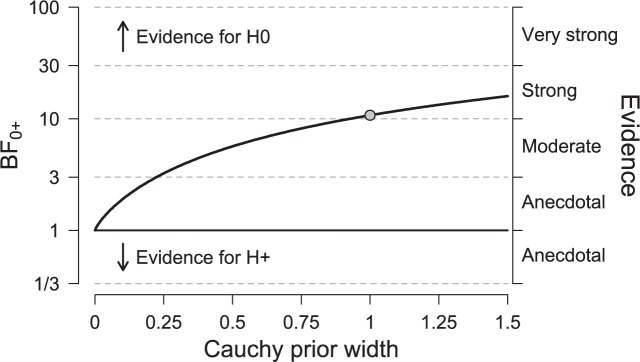
**Bayes factor as a function of the scale parameter *r* of the Cauchy prior for effect size under the alternative hypothesis**. Equal variances are assumed. The dot indicates the result from the default prior. Figure adjusted from JASP, jasp-stats.org.

To examine the robustness of our conclusions, we varied the shape of the prior for the effect size under the alternative hypothesis. **Figure 4** shows the Bayes factor as a function of the scale parameter *r* of the JZS Cauchy prior. The dot indicates the result from the default prior used in the preregistered data analysis (i.e., *r*= 1, as proposed by Jeffreys, 1961, and Rouder et al., 2009). It is evident that, as the scale parameter *r* increases (i.e., the prior becomes progressively wider), the Bayes factor increasingly favors the null hypothesis. In addition, it is evident that, even under the prior setting that most favors the alternative hypothesis (i.e., scale parameter *r* very close to zero), the Bayes factor is close to one, indicating ambiguous evidence.

### Exploratory Analyses 4: Additional Measures

The preregistration document reads: “As the original results from Topolinski and Sparenberg (2012) were not affected by the participants’ self-rated mood or arousal, we will not incorporate these factors in our confirmatory replication analysis (although we might present them later while explicitly acknowledging their exploratory nature).” Here we briefly describe the effects of the additional control factors.

The control factors were pleasantness (“How pleasant did you find this task?”), effort (“How much effort did you invest in this task?”), mood (“At this moment, you do you feel?”), and arousal (“At this moment, how agitated are you?”). These factors were assessed by Likert scales ranging from 0 to 10. **Table 1** shows the results separately for each condition. The two-sided default JZS Bayes factors indicate evidence in favor of the null hypothesis, with the exception of the “effort” control factor, for which the evidence is almost perfectly ambiguous; hence, these Bayes factors provide little encouragement to put forward any of these control factors as post-hoc explanations for our main result.

**Table 1:** Number of participants (N), mean and SD of openness to experience, and the two-sided default Bayes factors for each of the four control questions.

	Condition	*N*	Mean score	SD	BF_01_
Pleasantness	Clockwise	48	3.88	2.58	6.50
	Counterclockwise	54	3.81	2.06	
Effort	Clockwise	48	3.60	2.37	0.98
	Counterclockwise	54	4.56	2.37	
Mood	Clockwise	48	6.33	1.52	3.52
	Counterclockwise	54	5.94	1.85	
Arousal	Clockwise	48	3.06	1.73	2.42
	Counterclockwise	54	3.69	2.46	

## Discussion

We were unable to replicate the finding reported by Topolinski and Sparenberg (2012, Experiment 2). Based on data from 102 participants, our preregistered Bayes factor hypothesis test revealed that the data were 10.76 times more likely under the null hypothesis than under the alternative hypothesis. The observed effect size was slightly negative, indicating that the effect went in the direction opposite to that predicted by Topolinski and Sparenberg (2012). In sum, the results of our experiment do not support the idea put forward by Topolinski and Sparenberg (2012) that clockwise movements induce psychological states of temporal progression and an orientation toward the future and novelty.

We hope that future empirical efforts in psychology and other disciplines will increasingly use preregistered Bayes factor hypothesis tests. By preregistering the analysis plan, researchers prevent themselves from falling prey to their own preconceptions and biases, mental distortions that can easily translate in a series of data-inspired hypothesis tests, only a subset of which is presented to the reader. By conducting a Bayesian hypothesis test –something that can be easily accomplished using JASP ^3^ (Love et al., 2015), a free and open source graphical user interface for common statistical analyses– researchers can quantify and monitor evidence in favor of the null hypothesis and the alternative hypothesis.

In closing, we should stress that a single experiment cannot overturn a large body of work. However, the strength of evidence in our data is sufficient to change one’s prior beliefs by an order of magnitude. An empirical debate is best organized around a series of preregistered replications, and perhaps the authors whose work we did not replicate will feel inspired to conduct their own preregistered studies. In our opinion, science is best served by ruthless theoretical and empirical critique, such that the surviving ideas can be relied upon as the basis for future endeavors. A strong anvil need not fear the hammer, and accordingly we hope that preregistered replications will soon become accepted as a vital component of a psychological science that is both though-provoking and reproducible.

## Conflict of Interest Statement

The authors declare that the research was conducted in the absence of any commercial or financial relationships that could be construed as a potential conflict of interest.

## Footnotes

1 https://osf.io/8mpji/wiki/home/

2 www.test.uva.nl

3 https://jasp-stats.org
